# Section on General Medicine

**Published:** 1900-03

**Authors:** Henry P. Cooke, S. C. Red

**Affiliations:** Chairman; Galveston, Texas; Secretary, Houston, Texas


### TEXAS STATE MEDICAL ASSOCIATION.

### Section on General Medicine.

---

GALVESTON, TEXAS, February 8, 1900.

DEAR DOCTOR: The Texas State Medical Association meets at Waco, Texas, on Tuesday, the 24th of next April, and there is every reason to expect a full attendance. It is especially desirable that the

Section on General Medicine shall be well represented, and its work fully up to the standard of excellence maintained in the past.

With this end in view you are invited to prepare a paper, upon a subject of your own choosing, to be presented in this section.

To insure publication in the general program, the title of your paper, and the names of two members selected by you to lead the discussion, should be sent to the secretary not later than March 15th, next.

Very truly yours,

HENRY P. COOKE, M. D., Chairman.

S. C. RED, M. D., Secretary, Houston, Texas.

